# Clinical Gynecology, Medical and Surgical

**Published:** 1895-12

**Authors:** 


CLINICAL GYNECOLOGY, MEDICAL AND SURGICAL, for Students and Practitioners by Eminent American Teachers. Edited by John M. Keating, M. D., LL. D., and by Henry C. Coe, M. D., M. R. C. S., Professor of Gynecology, New York Polyclinic. In one octavo volume of 994 pages. Illustrated with 34 plates and nearly four hundred figures. Price, cloth, $6.00; sheep, $7.00; half Russia, $8.00. J. B. Lippincott Company, Publishers, Philadelphia. 1895.

In the preparation of this work the plan was adopted to eliminate the larger portion of the theoretical part of gynecology, and make this differ from the ordinary works on this subject in that this one contains more of the purely practical, teaching, making of this work a clinical teacher, in reality serving as a post-graduate course of clinical instruction on the subject of gynecology. It will be seen, then, that this volume is intended for the practicing physician rather than the medical student. The contributors to this volume were selected by Dr. Keating because of their especial fitness for the work assigned them, and the completed volume represents the combined experiences of a number of our most prominent teachers of gynecology. The list includes Drs. William H. Baker, Francis H. Davenport, Hunter Robb, Bache McE. Emmet, Barton C. Hirst, Matthew D. Mann, William M. Polk, J. Whitridge Williams, Henry T. Byford, Paul F. Mundé, Hermann J. Boldt, Henry C. Coe, William T. Lusk, Chauncey D. Palmer, Charles Jewett, John Polak, Edward E. Montgomery,

Dudley P. Allen, and a chapter on cutaneous diseases peculiar to women by Drs. Louis A. Duhring and Milton B. Hartzell.

The introductory was written by Dr. William Goodell, and as the last words of this gifted teacher, it will be read by very many with mournful interest.

This work will meet with the approbation of a large number of the profession, and as a clinical work on the subject of gynecology it stands without an equal in this country.

The mechanical work, paper, binding and illustrations, are all excellent, the latter being numerous, and most of them original.

